# Supplementation in vitamin B3 counteracts the negative effects of tryptophan deficiencies in bumble bees

**DOI:** 10.1093/conphys/coac084

**Published:** 2023-01-23

**Authors:** M L Tissier, S Kraus, T Gómez-Moracho, M Lihoreau

**Affiliations:** Biological Sciences, Bishop’s University, 2600 Rue College, Québec J1M 1Z7, Canada; Research Center on Animal Cognition, Center for Integrative Biology; CNRS, University Paul Sabatier, 31062 Toulouse, France; Research Center on Animal Cognition, Center for Integrative Biology; CNRS, University Paul Sabatier, 31062 Toulouse, France; Research Center on Animal Cognition, Center for Integrative Biology; CNRS, University Paul Sabatier, 31062 Toulouse, France

**Keywords:** nutrition, nicotinamide, niacin, essential amino acids, Bumblebee

## Abstract

Increasing evidence highlights the importance of diet content in nine essential amino acids for bee physiological and behavioural performance. However, the 10th essential amino acid, tryptophan, has been overlooked as its experimental measurement requires a specific hydrolysis. Tryptophan is the precursor of serotonin and vitamin B3, which together modulate cognitive and metabolic functions in most animals. Here, we investigated how tryptophan deficiencies influence the behaviour and survival of bumble bees (*Bombus terrestris*). Tryptophan-deficient diets led to a moderate increase in food intake, aggressiveness and mortality compared with the control diet. Vitamin B3 supplementation in tryptophan-deficient diets tended to buffer these effects by significantly improving survival and reducing aggressiveness. Considering that the pollens of major crops and common plants, such as corn and dandelion, are deficient in tryptophan, these effects could have a strong impact on bumble bee populations and their pollination service. Our results suggest planting tryptophan and B3 rich species next to tryptophan-deficient crops could support wild bee populations.

## Teaser

While many bumble bee species are threatened, information on their nutritional ecology is still scarce, especially regarding the role of dietary micronutrients. Here, we show that deficiencies in the essential amino acid tryptophan impair bumble bee behaviour and survival but that a supplementation in vitamin B3 counteracts these effects.

## Introduction

Insect pollinators, including wild bees, are declining worldwide (Klein *et al.*, 2017; Didham *et al.*, 2020; Wagner, 2020). This is exemplified by bumble bees, which provide important natural and crop-related pollination services, but have declined in Northern America and Europe, with important inter-specific variations in the observed trends (Cameron *et al.*, 2011; Guzman *et al.*, 2021; Jackson *et al.*, 2022). Causes of such declines are multiple and include habitat loss, climate change, pesticides and disease spillover from managed bees (Goulson *et al.*, 2015; Cameron and Sadd, 2020).

Recently, there has been a growing interest in the possibility of malnutrition being a major cause of bee decline (Wright *et al.*, 2018). The generalization of crop monocultures constrains pollinators to suboptimal monotonous diets (Goulson *et al.*, 2015; Klein *et al.*, 2017). For bees that rely on carbohydrates in nectar, and proteins, minerals, vitamins and lipids in pollen (Brodschneider and Crailsheim, 2010; Vaudo *et al.*, 2016), inappropriate nutrient intake can strongly impair their physiology (Di Pasquale *et al.*, 2013; Conroy *et al.*, 2016), survival (Conroy *et al.*, 2016) and colony growth (Moerman *et al.*, 2017). Recent studies show that the concentration and balance of key nutrients in nectars and pollens [e.g. content in essential amino acids, EAAs (Leonhardt and Blüthgen, 2012; Vanderplanck *et al.*, 2014; Stabler *et al.*, 2015; Moerman *et al.*, 2016b, 2017) or omega 6:3 ratio (Arien *et al.*, 2015)] influence many parameters related to bee performances, such as olfactory and tactile associative learning (Arien *et al.*, 2015), egg production (Vanderplanck *et al.*, 2014) and larval development (Vanderplanck *et al.*, 2014; Moerman *et al.*, 2016b, 2017). Regarding protein quality, studies are increasingly looking at the total concentration in total amino acids or EAAs (Leonhardt and Blüthgen, 2012; Vanderplanck *et al.*, 2014; Stabler *et al.*, 2015; Moerman *et al.*, 2016b, 2017; Ryder *et al.*, 2021) as an index of diet quality to which colony growth has been associated (Leonhardt and Blüthgen, 2012; Moerman *et al.*, 2016b, 2017).

So far one EAA has been overlooked, tryptophan (Leonhardt and Blüthgen, 2012; Vanderplanck *et al.*, 2014; Moerman *et al.*, 2016b, 2017; Kriesell *et al.*, 2017; Ryder *et al.*, 2021), since its experimental measurement requires a specific alkaline hydrolysis, while all others EAAs necessitate an acid hydrolysis (Standifer *et al.*, 1980). Tryptophan is not only necessary for protein and enzyme syntheses but is also essential to produce serotonin, melatonin and vitamin B3 (de Arruda *et al.*, 2013; Kohlmeier, 2015). Converted into serotonin or melatonin, tryptophan modulates many functions, including appetite, sleep and aggressive behaviour in most species studied (Bubak *et al.*, 2020). Tryptophan is also involved in cell respiration and ATP synthesis through its conversion into vitamin B3, i.e. generic name of nicotinic acid and nicotinamide (Penberthy and Kirkland, 2020). Nicotinamide is the precursor of the coenzyme nicotinamide adenine dinucleotide (NAD), which plays a crucial role in the Krebs cycle and modulates the function of hundreds of enzymes (Kohlmeier, 2015; Penberthy and Kirkland, 2020).

Tryptophan is solely produced by plants and micro-organisms (Kohlmeier, 2015). Animals are unable to synthetize it, and most of them have a reduced capacity to transform it into vitamin B3 (Baker, 2008; Kohlmeier, 2015). In humans, a lack of tryptophan in the diet causes Pellagra, a disease characterized by a variety of symptoms, including dementia, diarrhoea and dermatitis (i.e. the 3Ds disease) (Hegyi *et al.*, 2004; Wan *et al.*, 2011). In animal models, such as mice and rats, tryptophan deficiencies lead to growth retards and high rates of aggressiveness (Krehl *et al.*, 1945; Kantak *et al.*, 1980; Walz *et al.*, 2013). However, supplementations in vitamin B3 is used to cure Pellagra in humans (Penberthy and Kirkland, 2020) and shows important properties in the treatment of cognitive disorders or age-related pathologies (cancer and glaucoma) in mice (Williams *et al.*, 2017; Sarkar *et al.*, 2020). Similar effects were also observed in non-model animals, such as in the endangered common hamster where dietary vitamin B3 supplementation reduces infanticides caused by tryptophan deficient corn diet (Tissier *et al.*, 2017).

Although there is an abundant literature in vertebrates (Krehl *et al.*, 1945; Meisinger and Speer, 1979; Kantak *et al.*, 1980; Hegyi *et al.*, 2004; Baker, 2008; Walz *et al.*, 2013; Tissier *et al.*, 2017), studies of these effects on invertebrates are scarce (de Groot, 1953; Bubak *et al.*, 2020; Csata *et al.*, 2020). Early work indicates tryptophan deficiencies reduce growth and survival in the Western honeybee (de Groot, 1953). A diet content of 12 mg of tryptophan per gram of food is considered optimal in honey bees, maximizing workers food intake, antioxidant capacity, serotonin concentration, hypopharyngeal gland acinus size (the primary organ secreting jelly), body weight and lifespan. Below and above this concentration, detrimental effects were recorded, such as the reduction of food intake and lifespan (Fengkui *et al.*, 2015). While this is an important first step, virtually nothing is known on the effects of deficiencies in tryptophan or its derivates on other major pollinators in the context of widespread decline. Tissues of many common plants, including pollen, are known to be deficient in tryptophan and to contain a bounded form of vitamin B3, non-bioavailable to animals. These include most cereals and some weeds, namely corn and dandelion, whose pollen is reported in the diet of a diversity of bee species in spring and summer (Auclair and Jamieson, 1948; Brian, 1951; Hogan *et al.*, 1955; Henderson *et al.*, 1959; Goss, 1968; Teper, 2006; Danner *et al.*, 2014; Requier *et al.*, 2015; Di Pasquale *et al.*, 2016; Ammerman *et al.*, 1995). Understanding the consequences of tryptophan deficiencies on wild pollinators is therefore critical to better manage environmental resources available to them, with the aim of increasing the efficiency of conservation plans and pollination practices.

Here, we investigated how tryptophan deficiencies mimicking those found in corn or dandelion pollen (Auclair and Jamieson, 1948; Goss, 1968) influenced food intake, lifespan and aggressiveness in the buff-tailed bumble bee (*Bombus terrestris*), a major pollinator of crops and wild plants. We exposed micro-colonies of bumble bees to sucrose-based solutions varying in their tryptophan and vitamin B3 content. We predicted that tryptophan deficiencies in the diet would reduce food intake and longevity, and increase aggressiveness, while supplementation in vitamin B3 should buffer these effects.

## Materials and Methods

### Bumble bees

We purchased four *B. terrestris* colonies (BioBest, Belgium) from which we built 60 micro-colonies. Each of these micro-colony was composed of 10 workers of unknown age randomly collected in the four mother colonies (N = 600 workers). All workers were marked with coloured numbered tags on the thorax for individual identification. Micro-colonies were maintained in bipartite plastic boxes [10 × 16 × 16 cm; see (Kraus *et al.*, 2019)] under controlled conditions: temperature 25°C–27°C; humidity 35%–45%; photoperiod 12 L: 12 D. Bumble bees were fed *ad libitum* with 50% (w/w) sucrose solution supplemented in the 10 essential amino acids (de Groot, 1953) as described below.

### Artificial diets

Micro-colonies were fed one of four artificial diets (15 micro-colonies/diet and 150 workers/diet): a control diet and three treatment diets with varying concentrations of tryptophan and nicotinamide (Table 1). The control diet was prepared based on previously measured consumptions of *Rubus* pollen by bumble bees [see Table S2 in Di Pasquale *et al.* (2013)]. *Rubus* is a valuable monofloral source of pollen for honey bees rich in protein and whose content in EAAs matches that of pollens collected by *B. terrestris* (Leonhardt and Blüthgen, 2012; Vanderplanck *et al.*, 2014; Kriesell *et al.*, 2017). Assuming that *B. terrestris* workers assimilate ~20% of ingested amino acids from ingested proteins (Stabler *et al.*, 2015) and that their daily intake of pollen under captive conditions is of 0.3-g maximum (Vanderplanck *et al.*, 2014; Moerman *et al.*, 2017), the estimated daily requirement of each EAA was estimated to match the content in each essential amino acid of *Rubus spp.* pollen as described in Table S2 of (Di Pasquale *et al.*, 2013). As EEA content was provided in g/100 g of pollen, we multiplied the value by 10 to obtain a value in mg/g. Thus, the daily requirement of workers was assessed as follows:

Eq. 1:


Eq. 1: \begin{align*} &Daily\ requirement\ of\ each\ EAA\ \Big(\frac{mg}{day}\Big)\nonumber\\&=0.2\times \Big({EAA}_{Content\ \left(\frac{g}{100g}\right)}\Big)\times 10\times 0.3\nonumber \end{align*}

**Table 1:** Concentrations of essential amino acids (in mg/mL) in the diets

Diet		Control reference (*Rubussp*.)	Control	MT	LT	LTN
EAAs	Unit	mg/day	mg/mL	mg/mL	mg/mL	mg/mL
Valine		0.684	0.684	0.684	0.684	0.684
Methionine		0.324	0.324	0.324	0.324	0.324
Isoleucine		0.546	0.546	0.546	0.546	0.546
Leucine		0.888	0.888	0.888	0.888	0.888
Threonine		0.354	0.354	0.354	0.354	0.354
Phenylalanine		0.594	0.594	0.594	0.594	0.594
Lysine		0.9	0.9	0.9	0.9	0.9
Histidine		0.276	0.276	0.276	0.276	0.276
Arginine		0.618	0.618	0.618	0.618	0.618
Tryptophan		0.162	0.162	0.005	0.0001	0.0001
Nicotinamide		/	/	/	/	0.006

Estimated daily consumption of each of the 10 EEAs by bumble bee workers if fed on *Rubus* pollen, based on its composition (Di Pasquale *et al.*, 2013) and daily intake and assimilability of EAAs from pollen (Leonhardt and Blüthgen, 2012; Vanderplanck *et al.*, 2014; Stabler *et al.*, 2015; Kriesell *et al.*, 2017). Control = control diet mimicking the composition of *Rubus* pollen, LT = low tryptophan (mimicking what is found in corn and dandelion pollen, i.e. traces of trp), MT = medium tryptophan (intermediate between the LT and control diets) and LTN = low tryptophan with nicotinamide supplementation.

Diets were prepared in a final volume of 500 mL. They consisted in 50% sucrose solution (w/w) with 0.5% of insect vitamin cocktail (Sigma, Germany), plus the corresponding EAA (Table 1). In order to adjust low EAA concentrations, tryptophan was added to MT, LT and LTN diets from an intermediate solution at 0.05 mg/mL of tryptophan in distilled water. Nicotinamide in LTN was added from a solution of 3 mg/mL of nicotinamide in distilled water. Diets were provided in a gravity feeder consisting of a 15-mL tube with two holes at its basis, allowing bumble bees to insert their proboscis and ingest the solution.

### Food intake and survival

We let the bumble bees acclimate to the micro-colony setup during 6 days. We then monitored the food intake of the workers by renewing all diets every 2 days with a new cup to collect potential leakage (Kraus *et al.*, 2019). Each vial and cup were weighed before and after being provided to the workers from Day 7 (first behavioural trial) to Day 18 (end of the experiment), using a precision balance of 1 mg (ME103T; Mettler Toledo, Switzerland). Evaporation or leakage of the sucrose solution was estimated by placing six gravity feeders in separate locations (without any bees) in the room and weighing them every 2 days.

Survival was assessed on bumble bee workers from 40 micro-colonies (*n* = 10/diet). Dead bumble bee workers were recorded and removed daily in each micro-colony. The estimation of the evaporation and leakage of the sucrose solution assessed in the absence of bees together with the number of living workers in each micro-colony were used to assess food intake using the formulas described below, a standard approach used in experiments conducted on queenless micro-colonies (Kraus *et al.*, 2019; Giacomini *et al.*, 2022).

Calculation of daily food intake (intake of the sucrose solution in g.bee.day^−1^) and cumulative food intake per bee throughout the experiment was conducted following (Kraus *et al.*, 2019). We considered food collected as food ingested, given that there is no trophallaxis in bumble bees and that there was no honey pot through which the sucrose solution could be stored in our micro-colonies (Liebig *et al.*, 1997; Dornhaus and Chittka, 2005).

Eq. 2:


Eq. 2: \begin{equation*} Initial\ Diet= Diet\ weight\ before\ Test- Mean\ Tube\ weight\nonumber \end{equation*}

$$ \begin{align*} Remaining\ diet&=\! Diet\ weight\ after\ Test-\! Mean\ Tube\ weight\\&+ Cup\ weight\ after\ Test- Mean\ Cup\ weight \end{align*}$$

Measure of leakage or evaporation (assessed in the six feeders without bees):

$$ \small\begin{align*} Leakage\ \left(\% per\ hour\right)=\frac{\left( Initial\ Diet- Remaining\ Diet\right)}{Initial\ Diet\ast Time}\ast 100 \end{align*}$$

Amount of food ingested:

$$ \small\begin{align*} &Daily\ Food\ Intake\ per\ Bee\\&\quad=\frac{Initial\ Diet\ast \left(1- Mean\ Leakage\ast Time/100\right)- Remaining\ Diet}{Number\ of\ Bee} \end{align*}$$

$$ \begin{align*} &Cumulative\ Food\ Intake\ per\ Bee\\&\quad={\sum}_{i=0}^{Day} Daily\ Collected\ Diet\ per\ Bee\ at\ Day\ i \end{align*}$$

### Behavioural interactions and aggressive behaviour

**Figure 1:**
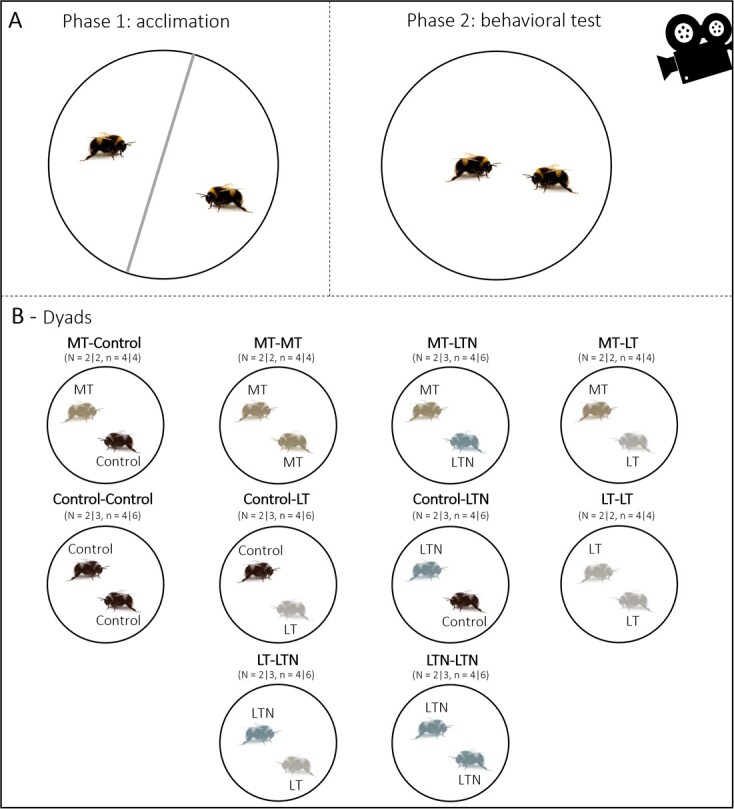
The optimal content, based on *Rubus spp.* pollen, was provided to the control group. The diet content of the nine EAAs was identical between the three experimental diets (LT, MT, LTN) and the control diet. Only the tryptophan content varied, with a medium (MT) or reduced (LT, LTN) content. The LTN diet thus had an identical EAAs composition to the LT diet but was supplemented in nicotinamide (Table 1). Workers of *B. terrestris* ingest on average 1 mL of control diet per day in our laboratory conditions (preliminary data not shown) that matches the estimated daily intake in EAAs if bumble bees were fed *Rubus* pollen. Experimental design used to assess workers behaviour. In (A), the two phases of the behavioural test are shown. Phase 1 corresponds to the 5-minute acclimation period with a plastic separator preventing workers to interact. Phase 2 corresponds to the 5-minute video-taped behavioural trial, during which workers were allowed to interact in the petri dish. In (B), the 10 dyads tested are represented. Numbers of dyads (N) and number of workers (n) tested per diet are shown for each trial, respectively (first | second). A total of 92 bumble bees were tested (40 in trial 1 and 52 in trial 2, 21–25 bees/diet). The same colour code is used in Figs 1–4.

Behavioural assays were conducted on individuals from 20 of the 60 micro-colonies (five micro-colonies/diet) on Days 7 and 15. The delay of 8 days was chosen to have two behavioural independent trials and to focus on the impact of the diet on individual behaviour. This delay reduces the risk that the behavioural response of a bee tested in Test 2 will be influenced by the behaviour of its nestmate when returning to the colony after Trial 1. We quantified aggressiveness in dyads of individuals (10 different dyads; Fig. 1). Each trial consisted of a 5-minute acclimation period during which two bumble bees were placed in a petri-dish (10 cm diameter) divided in two chambers with a plastic separator. After this period, the separator was removed to allow the bumble bees to interact. Each trial was videotaped during the 5 minutes (Sony Handycam Flash HDR-CX240EB), which started at the removal of the plastic separator. We tested all 10 diet combinations and obtained two to three replicates for each combination (see Table S1). We recorded and differentiated total physical and aggressive interactions between workers. Physical interactions included all occurrences with body contact (antennation, head or abdomen contact) between the two workers. Aggressive interactions included biting and body contacts with opened mandibula, which are considered as extreme expressions of aggression, i.e. attacks (Duchateau, 1989; Amsalem and Hefetz, 2011; Padilla *et al.*, 2016). After each trial, workers were placed back in their respective micro-colonies.

**Figure 2:**
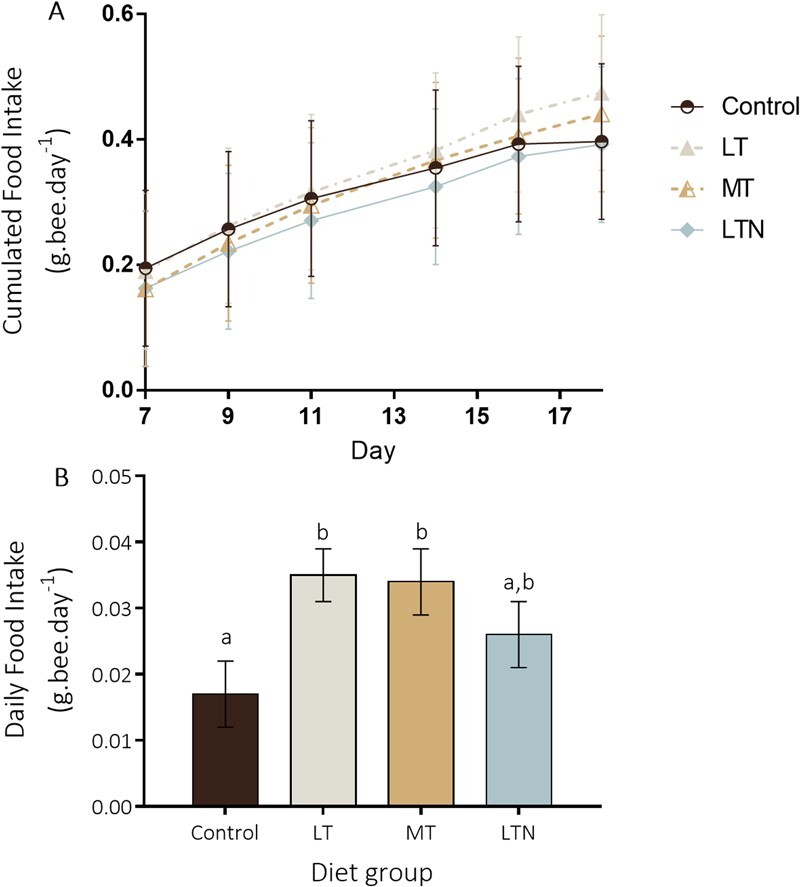
Food intake. (A) Individual cumulated food intake (in g.bee.day-1) from Day 7 to Day 18 and (B) individual daily food intake on Day 18. Control diet (circles), LT = low-tryptophan (filled triangles), MT = medium-tryptophan (half-filled triangles) and LTN = low-tryptophan with nicotinamide supplementation (filled diamonds). Different letters represent significant differences between the groups (LM, *P* < 0.05). Means ± SEM are represented. The same colour code is used in Figs 1–4.

### Statistical analyses

*Food intake.* We tested the diet effect on the cumulated food intake from Day 7 to Day 18 using a linear mixed model (LMM). We included the diet, the day and the diet*day interaction as fixed effects and the micro-colony ID as a random effect to control for repeated measures on the same micro-colonies. We also investigated the effect of the diet on the daily food intake (g.bee.day^−1^) on the last day of the experiment (daily food intake on Day 18) using a linear model (LM) with the diet as fixed effect.

*Survival.* We conducted a Cox’s proportional hazard model to test the effect of the diet on workers survival from Day 0 to Day 18. This was conducted using workers from the 40 micro-colonies where survival was monitored (see above). There was a relatively high mortality (41%) in all diet groups in the first 24 hours of the experiment, likely because of handling stress following first tagging. Workers that died during this timeframe were replaced with new individuals, only in the 40 colonies used for the survival analyses. Analyses were conducted both including and excluding workers that died during the first 24 hours.

*Interactions and aggressive behaviour.* We used LMMs to investigate diet effects on the number of interactions and number of attacks between workers during the behavioural trials. We included the diet, the date of trial and the interaction between these two variables as fixed effects. The micro-colony ID and colony of origin were added as random effects to control for repeated measures on the same micro-colonies and for potential genetic effects on aggressiveness (i.e. colony of origin). We initially estimated that a minimum of 10 repetitions/dyad or combinations would be necessary to assess a dyad effect. However, due to the high mortality of workers during the first 48 hours of the study and considering that we did not have enough bumble bees to replace all the workers in the 20 micro-colonies destined to behavioural tests, we ended with two to three repetitions per dyad (Fig. 1). We thus could not test for a dyad*diet interaction effect (e.g. whether control bumble bees reacted differently when confronted to LT, MT or LTN bumble bees).

In all LMs, normality of the residuals was tested using a Kolmogorov–Smirnov test and homogeneity of variances using a Bartlett test. Analyses were conducted using SPSS software (IBM SPSS Statistics for WINDOWS v. 24.0. Armonk, NY: IBM Corp.). Data presented are means ± SEM except when specified otherwise.

## Results

1. Tryptophan deficiencies increased food intake

We found no significant effect of the diet on the average individual cumulated food intake between Day 7 and Day 18 (LMM, F_3;51.5_ = 0.44, *P* = 0.72; Fig. 2A). However, there was an effect of the diet on the average individual daily food intake on Day 18 (LM, F_3;31_ = 3.18, *P* = 0.038; Fig. 2B). On the last day of the experiment, bumble bees from the LT and MT diet groups ingested significantly more food than bumble bees from the control diet group (Fig. 2B, mean difference of 0.018 ± 0.006 and 0.017 ± 0.007, respectively, t = 2.72, *P* = 0.011 and t = 2.57, *P* = 0.015). Bumble bees from the LTN diet group did not ingest significantly more or less food than the other diet groups (average daily food intake on Day 18: C = 0.017 ± 0.005, LT = 0.035 ± 0.004, MT = 0.034 ± 0.005, LTN = 0.026 ± 0.005 g.bee.day^−1^, *P* > 0.05).

2. Tryptophan deficiencies decreased survival

**Figure 3:**
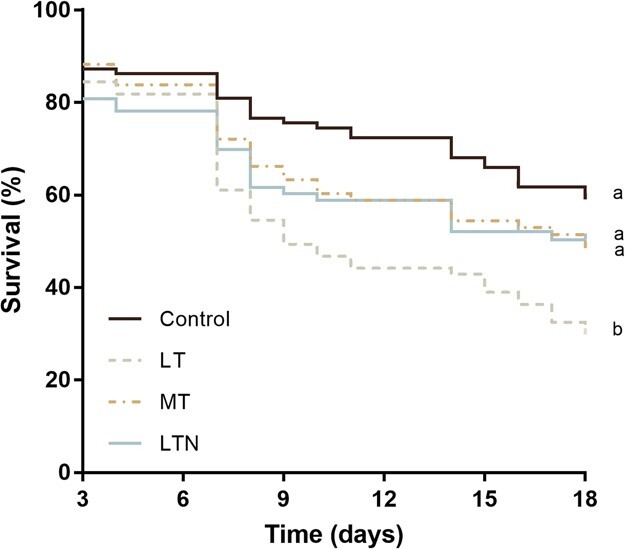
Survival. Survival rates are shown between Day 3 and Day 18 (N = 312 workers), considering the high mortality that occurred during the first 48 hours of the study and the replacement of workers (survival rates for the entire period are shown on Fig. S1). Different letters indicate significant differences between the diet groups (Cox model, *P* < 0.05). Control, LT = low-tryptophan, MT = medium-tryptophan and LTN = low-tryptophan with nicotinamide supplementation. The same colour code is used in Figs 1–4.

We found a diet effect on survival (Cox model; Fig. 3). Workers fed the control diet had twice the survival probability of workers fed the LT diet (control vs LT: β = 0.75 ± 0.21, *P* < 0.001, exp(β) = 2.11, 95% CI = 1.39–3.19). Workers fed the LTN and MT diets also had a significantly greater survival probability than workers fed the LT diet (LTN vs LT: β = 0.43 ± 0.21, *P* = 0.047, exp(β) = 1.53, 95% CI = 1.01–2.34; and MT vs LT: β = 0.43 ± 0.22, *P* = 0.047, exp(β) = 1.54, 95% CI = 1.01–2.36). However, workers fed MT and LTN diets displayed survival probability that did not differ from that of those fed the control diet (LTN vs control: β = −0.32 ± 0.23, *P* = 0.17, exp(β) = 1.38, 95% CI = 0.46–1.15; MT vs control: β = −0.31 ± 0.23, *P* = 0.18, exp(β) = 0.73, 95% CI =0.46–1.16) or from each other (MT vs LTN: β =0.01 ± 0.24, *P* = 0.98, exp(β) =1.01, 95% CI = 0.63–1.60). This analysis was conducted without including the data of first 48 hours since many workers died and were replaced with new ones (see Material and Methods). Including the data from the first 48 hours provided similar results with an intermediate survival rate for the LTN group (Fig. S1 and Supplementary Results).

3. Tryptophan deficiencies increased aggressiveness

**Figure 4:**
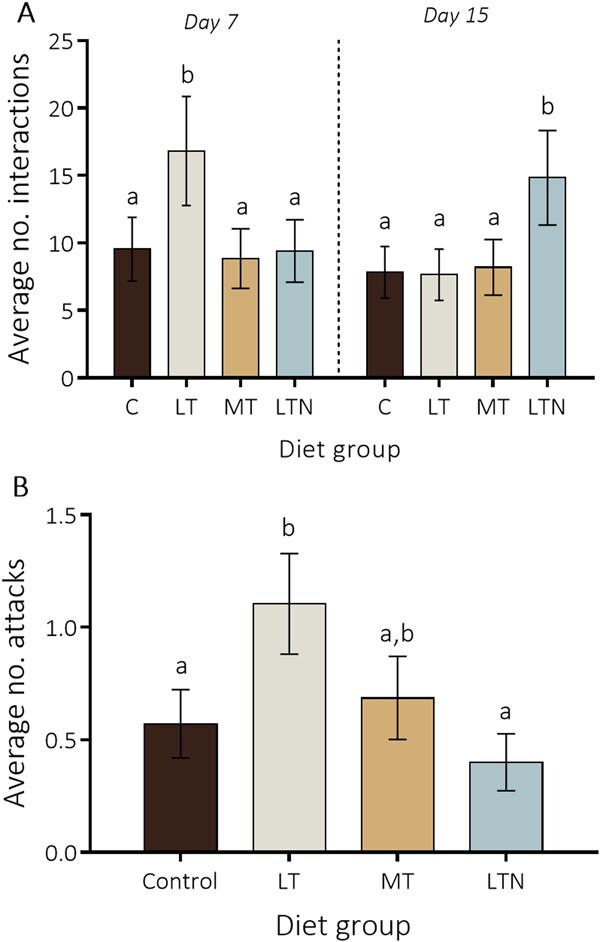
Behaviour. (A) Average number of physical interactions recorded between workers at Day 7 (*n* = 40) and Day 15 of the experiment (*n* = 50, among which 10 dyads were tested on Day 16). (B) Average number of attacks recorded from workers fed a given diet. LT = low-tryptophan, MT = medium-tryptophan and LTN = low-tryptophan with nicotinamide supplementation. Different letters highlight a significant difference (LMM, *P* < 0.05) between the diet groups. Means ± SEM are represented. The same colour code is used in Figs 1–4.

We found a significant diet*period interaction (F_3;82_ = 16.329, *P* < 0.001) on the mean number of physical interactions between workers during the behavioural trials (Fig. 4A). On Day 7, bumble bees fed LT diet had significantly more interactions with their conspecifics than bumble bees fed the three other diets (*P* < 0.004; Fig. 4A). In contrast, on Days 15 and 16, the mean number of physical interactions was significantly greater in bumble bees fed LTN diet than in bumble bees fed the other diets (*P* = 0.001; Fig. 4A). This could be related to the expression of defensive behaviours by some of the LTN fed workers when exposed to MT and LT fed workers, which were observed in those trials. These defensive behaviours included ‘prostrate’ (one bumble bee is lying on the side, while another bumble bee is involved in an aggressive interaction or an attack) and ‘escape’ (one bumble bee is moving fast in an opposite direction/to create distance when another bumble bee is following or attacking). However, their appearance was too scarce for us to conduct statistical analyses.

We also found a significant effect of diet on the number of attacks (F_3;82_ = 3.017, *P* = 0.035) and an absence of effects of the period (F_1;82_ = 2.070, *P* = 0.154) and the interaction between these two variables (F_3;82_ = 0.788, *P* = 0.504). Bumble bees fed LT diet expressed significantly more attacks than bumble bees fed control and LTN diets (Fig. 4B; *P* = 0.035 and *P* = 0.009, respectively). The average number of attacks expressed by bumble bees fed MT diet did not differ significantly from those expressed by bumble bees fed LTN, LT and control diets (Fig. 4B; *P* = 0.12, *P* = 0.25 and *P* = 0.57). Tryptophan deficiencies thus increased aggressiveness among workers and this effect was counteracted by nicotinamide supplementation.

## Discussion

Previous studies showed a lack of several essential amino acids can have severe consequences on the reproductive and survival performances of bee species (Leonhardt and Blüthgen, 2012; Vanderplanck *et al.*, 2014; Stabler *et al.*, 2015; Moerman *et al.*, 2016b, 2017; Ryder *et al.*, 2021). However, tryptophan has not been evaluated because of experimental constraints. Here, we found a lack of tryptophan is detrimental in bumble bees, as reported in many vertebrates (Kantak *et al.*, 1980; Baker, 2008; Walz *et al.*, 2013; Tissier *et al.*, 2017). While tryptophan-deficient diets increased food intake, aggressiveness and mortality in bumble bees, supplementation of vitamin B3 partially buffered these effects on survival and behaviour.

### Dietary tryptophan deficiencies induced an increase in food intake, partially buffered by vitamin B3 supplementation

We found no significant effect of the diet treatment on the amount of food ingested throughout the experiment (Fig. 2A). However, there was a significant difference in the daily food intake at the end of the experiment between the diet groups. On Day 18, workers from the LT and MT diet groups ingested twice as much food than those from the control group. Workers from the LTN group did not significantly differ from the other groups, although they displayed daily food intake values that were intermediate between the control group and the two other experimental groups on Day 18 (Fig. 2B). These effects do not match what we initially predicted based on a previous study in honey bees, where a sub-optimal content in tryptophan in diet led to reduced food intake (Fengkui *et al.*, 2015). However, in that study, the authors sought to identify the optimal intake of tryptophan by the honey bee, using diets containing between 9 and 14 mg/g of tryptophan and composed of a mix between rape pollen and sucrose (Fengkui *et al.*, 2015). This is much higher than the tryptophan content of our LT and MT diets, where we sought to mimic the deficiencies found in corn and dandelion pollen, with tryptophan content ≤ 1 mg/g (Auclair and Jamieson, 1948; Goss, 1968; Hsu *et al.*, 2021). In our study, bumble bees may have slightly increased their daily food intake throughout the study, which was only measurable at Day 18, as a way to compensate for the dietary deficiency in tryptophan. This phenomenon was observed in ants and suggested to be a way to increase daily intake in tryptophan (Csata *et al.*, 2020). An alternative, but non-mutually exclusive, hypothesis is that bumble bee workers from the LT and MT diet may have increased their food intake on Day 18, just before dying, a phenomenon observed in honey bees (Bouchebti *et al.*, 2022). Finally, considering that tryptophan (as a precursor of serotonin) is known to control appetite (French *et al.*, 2014), the strong deficiency of tryptophan in the LT and MT diets may also have led to a loss in the down regulation of appetite and a consequent increase in food intake over time, cumulating at Day 18 in our experiment (French *et al.*, 2014). Since bumble bees rely less on nectar than honey bees and collect pollen with significantly higher content in amino acids (Leonhardt and Blüthgen, 2012), their daily requirements (optimum) and responses when below or above this optimum may vary, as well as the downregulation from serotonin on food intake.

### Tryptophan deficiencies reduced survival, which was counteracted by vitamin B3 supplementation

Tryptophan deficiencies reduced survival compared with the control group, as previously observed in honey bees fed a tryptophan-deficient diet (Fengkui *et al.*, 2015) or maize pollen (Velthuis, 1992; Höcherl *et al.*, 2012) known to be deficient in tryptophan (Goss, 1968). However, vitamin B3 supplementation significantly increased survival, so that workers fed the LTN diet had comparable survival rates to workers fed MT and control diets. This suggests the vitamin B3 supplementation of 0.006 mg/mL in sucrose solution was sufficient to promote survival levels like that of bumble bee workers fed control diets in our laboratory setting. However, given that the optimal tryptophan and vitamin B3 intakes for bees other than honey bees (Fengkui *et al.*, 2015) are unknown, future studies are needed to quantify precise daily intakes and correlate them with performance traits, especially for queens (e.g. survival and reproduction).

### Vitamin B3 supplementation counteracted the negative effects of tryptophan deficiency on aggressiveness

Tryptophan deficiencies also led to increased aggressiveness in worker bumble bees, with more attacks in workers fed LT diet compared with workers fed control and LTN diets. The vitamin B3 supplementation of 0.006 mg/mL in the sucrose solution increased the number of interactions between workers on Days 15 and 16 compared with the other diets and reduced the occurrences of aggressive interactions (attacks) between workers compared with those of the LT diet. This could be explained by the expression of defensive behaviours from bumble bees fed LTN, when confronted to bumble bees fed LT or MT diets, as occasionally observed during the second trials. Bumble bees fed LTN diet also displayed the lowest number of aggressive interactions when confronted to workers from other micro-colonies, which was twice as low as observed in the LT diet. These behavioural modifications might either be a consequence of modifications in the serotonin pathway or resulting from benefits of vitamin B3 on neuron health and functioning. Indeed, serotonin is known to modulate aggressiveness in many species (Pucilowski and Kostowski, 1983; Bubak *et al.*, 2020) and defensive behaviours in the honey bee (Hunt, 2007). Its synthesis could be related to diet content in vitamin B3 through tryptophan, which is a precursor of both molecules (de Arruda *et al.*, 2013; Kohlmeier, 2015). In addition, vitamin B3 is known to be essential for cell functioning and ATP synthesis, namely as the precursor of the synthesis of the coenzyme *NAD* (Wan *et al.*, 2011). In mammals, NAD serves as neural modulator and may regulate much behaviour, including anti-predatory behaviours (Richards *et al.*, 1983). The pathways by which tryptophan deficiency and vitamin B3 supplementation modulated the behaviour of bumble bees remain to be further investigated, but the role of these nutrients in modulating aggressiveness and social interactions thus appear essential. We therefore need ecologically driven studies on bee nutrition that will assess pollen content in tryptophan and vitamin B3 and how daily intakes of these nutrients can impact bee performance, as has been done for the nine other EAAs and other essential nutrients (Vanderplanck *et al.*, 2014; Arien *et al.*, 2015; Conroy *et al.*, 2016; Vaudo *et al.*, 2016; Moerman *et al.*, 2016b, 2017; Kriesell *et al.*, 2017; Ryder *et al.*, 2021).

### Ecological and fitness-related perspectives

Pollen of some widely distributed plants, such as corn and dandelion, are known to be deficient in tryptophan and some other essential amino acids for bees (Auclair and Jamieson, 1948; Goss, 1968). Honey bees fed maize or dandelion pollen showed strongly reduced survival rates as well as reduced rearing capacity and reproductive success compared with bees fed other pollen (Standifer *et al.*, 1980; Velthuis, 1992; Roulston and Cane, 2000; Höcherl *et al.*, 2012; Frias *et al.*, 2016). Consumption of maize pollen also impaired the development of the hypopharyngeal gland acini and reduced vitellogenin gene expression in nurses (Di Pasquale *et al.*, 2016). Furthermore, bumble bees fed dandelion pollen displayed very high rates of oophagy and larval ejection (100%), likely because of a deficiency in an essential amino acid (Génissel *et al.*, 2002). Although the direct link between tryptophan deficiencies and reduced performances has not been established in these studies, together with our findings, these observations echo what has been reported in a vertebrate, where corn consumption and associated tryptophan and vitamin B3 deficiencies led to abnormal behaviour, leading to 95% of maternal infanticides, as well as cannibalism, counteracted by vitamin B3 supplementation (Tissier *et al.*, 2017). Given the preponderance of dandelion and corn in terrestrial landscapes, this could have important ecological consequences for a wide diversity of bee species, known to collect these pollens (Brian, 1951; Teper, 2006; Requier *et al.*, 2015), by affecting not only their survival and aggressive behaviour but also their reproduction (Génissel *et al.*, 2002; Vanderplanck *et al.*, 2020). Recommendations to prevent the appearance of such deficiencies in corn and dandelion-dominated landscapes could involve the inclusion of vitamin B3 or tryptophan-rich plants. Although knowledge on the pollen content in these nutrients is rare, some plants with tryptophan-rich pollens seem to be good candidates, as for instance common sunflower, summer squash (*Curcubita pepo*), alfalfa (*Medicago sativa*) and broad beans (*Vicia faba*) (Yang *et al.*, 2013; Taha *et al.*, 2019). Sunflower was shown to be a proper food source to compensate for corn-related nutrient deficiencies in a vertebrate species (Tissier *et al.*, 2021). Interestingly, farmers following the Three Sisters, an agricultural cropping technique used by many first nations in North America, associated corn, squash and beans in the same system for their complementarity, already often use sunflower as a ‘fourth sister’ (Kapayou *et al.*, 2022). Poppies may offer another alternative of tryptophan-rich plants (Yang *et al.*, 2013). These plants are rich in tryptophan but do not flower in spring as dandelion does. Future research aimed at identifying recommendations for bee conservation should consider early flowering trees like maples and willows, as some species possess tissues that are especially rich in vitamin B3 (Burkholder and McVeigh, 1945) and appropriated for bee reproduction (Moerman *et al.*, 2016a,b; Yourstone *et al.*, 2021). Ultimately, the more detailed understanding of the nutritional requirements of bumble bees and other wild bees at the level of micronutrients will help better managing environmental resources made available to pollinators, for conservation and pollination purposes.

## Supplementary Material

Web_Material_coac084

## Data and materials availability

Data are available on OSF. Tissier Mathilde. 2022. ‘Data - Supplementation in vitamin B3 counteracts the negative effects of tryptophan deficiencies in bumble bees’. December 2. https://osf.io/qxsuy/.
