# Supplementary material for: Supplementation in vitamin B3 counteracts the negative effects of tryptophan deficiencies in bumble bees
Source: Conserv Physiol. 2023 Jan 23;11(1):coac084. doi: 10.1093/conphys/coac084 (PMC9871438; doi:10.1093/conphys/coac084)
Supplement: Web_Material_coac084 [file web_material_coac084.zip › REVISED_Supplementary_information_trp_vitaminB3_coac084.docx]

Supplementary information - Supplementation in vitamin B3 counteracts the negative effects of tryptophan deficiencies in bumblebees

Tissier M.L1, Kraus S., Gómez-Moracho T. and Lihoreau M.

**Supplementary results**

Survival analyses on the entire period (day 0 : installation of bees in microcolonies, to day 18: end of the experiment), showed a diet effect on subsequent worker survival (Cox model, Figure S1): workers from the LT and MT diet groups had reduced survival compared to workers from the control group (MT vs control: β = - 0.25 ± 0.11, p = 0.022, exp(β) = 0.78, 95% CI = 0.63-0.97 ; control vs LT: β = 0.26 ± 0.11, p = 0.016, exp(β) = 1.30, 95% CI = 1.05-1.61 ). LTN workers’ survival did not significantly differ from the LT, MT or control groups (LTN vs LT: β = -0.2 ± 0.11, p = 0.134, exp(β) = 0.85, 95% CI = 0.68-1.05, MT vs LTN: β = 0.02 ± 0.10, p = 0.882, exp(β) = 1.02, 95% CI = 0.83-1.25; control vs LTN: β = 0.16 ± 0.11, p = 0.140, exp(β) = 1.18, 95% CI = 0.95-1.47 ).

**
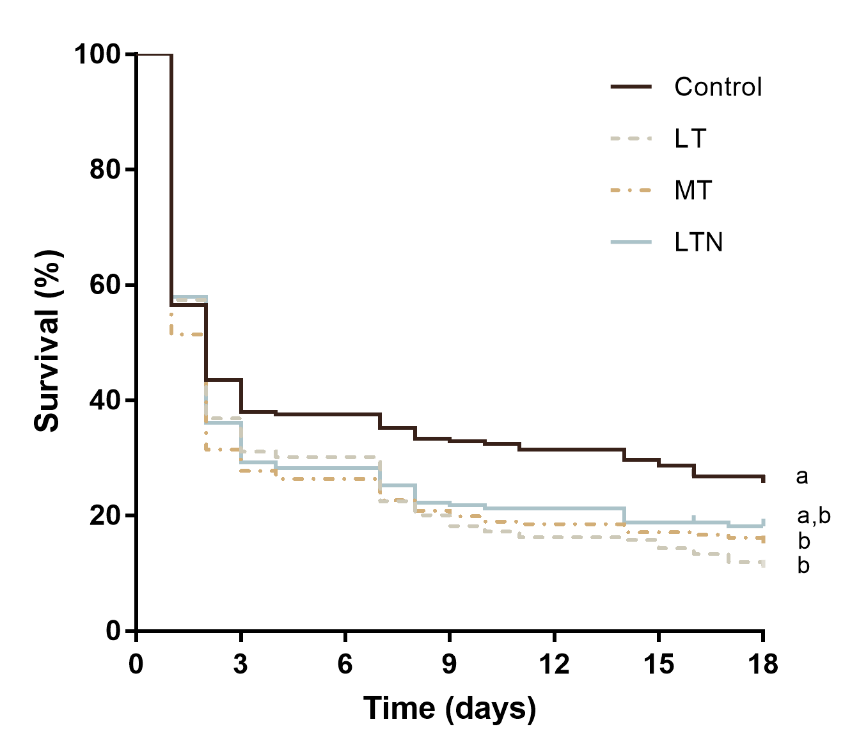
Figure S1. Diet effect on worker survival (%) over time (days) as a function of diet.** Survival rates are shown from day 0 to day 18, but bees were replaced on day 2 following a high mortality in all groups during the first 48 hours. Different letters highlight significant differences between the diet groups (Cox model, p<0.05). Control (a), LT = low-tryptophan (b), MT = medium-tryptophan (b) and LTN = low-tryptophan with nicotinamide supplementation (a, b).
